# Tetrathionate and Elemental Sulfur Shape the Isotope Composition of Sulfate in Acid Mine Drainage

**DOI:** 10.3389/fmicb.2017.01564

**Published:** 2017-08-17

**Authors:** Nurgul Balci, Benjamin Brunner, Alexandra V. Turchyn

**Affiliations:** ^1^Geomicrobiolog-Biogeochemistry Lab, Department of Geological Engineering, Istanbul Technical University Istanbul, Turkey; ^2^Department of Biogeochemistry, Max Planck Institute for Marine Microbiology Bremen, Germany; ^3^Department of Geological Sciences, University of Texas at El Paso El Paso, TX, United States; ^4^Department of Earth Sciences, University of Cambridge Cambridge, United Kingdom

**Keywords:** tetrathionate, oxygen isotopes, sulfur isotopes, microbial oxidation, intermediate valence state sulfur

## Abstract

Sulfur compounds in intermediate valence states, for example elemental sulfur, thiosulfate, and tetrathionate, are important players in the biogeochemical sulfur cycle. However, key understanding about the pathways of oxidation involving mixed-valance state sulfur species is still missing. Here we report the sulfur and oxygen isotope fractionation effects during the oxidation of tetrathionate (S_4_O_6_^2−^) and elemental sulfur (S°) to sulfate in bacterial cultures in acidic conditions. Oxidation of tetrathionate by *Acidithiobacillus thiooxidans* produced thiosulfate, elemental sulfur and sulfate. Up to 34% of the tetrathionate consumed by the bacteria could not be accounted for in sulfate or other intermediate-valence state sulfur species over the experiments. The oxidation of tetrathionate yielded sulfate that was initially enriched in ^34^S (ε^34^S_SO4−S4O6_) by +7.9‰, followed by a decrease to +1.4‰ over the experiment duration, with an average ε^34^S_SO4−S4O6_ of +3.5 ± 0.2‰ after a month of incubation. We attribute this significant sulfur isotope fractionation to enzymatic disproportionation reactions occurring during tetrathionate decomposition, and to the incomplete transformation of tetrathionate into sulfate. The oxygen isotope composition of sulfate (δ^18^O_SO4_) from the tetrathionate oxidation experiments indicate that 62% of the oxygen in the formed sulfate was derived from water. The remaining 38% of the oxygen was either inherited from the supplied tetrathionate, or supplied from dissolved atmospheric oxygen (O_2_). During the oxidation of elemental sulfur, the product sulfate became depleted in ^34^S between −1.8 and 0‰ relative to the elemental sulfur with an average for ε^34^S_SO4−S0_ of −0.9 ± 0.2‰ and all the oxygen atoms in the sulfate derived from water with an average normal oxygen isotope fractionation (ε^18^O_SO4−H2O_) of −4.4‰. The differences observed in δ^18^O_SO4_ and the sulfur isotope composition of sulfate (δ^34^S_SO4_), acid production, and mixed valence state sulfur species generated by the oxidation of the two different substrates suggests a metabolic flexibility in response to sulfur substrate availability. Our results demonstrate that microbial processing of mixed-valence-state sulfur species generates a significant sulfur isotope fractionation in acidic environments and oxidation of mixed-valence state sulfur species may produce sulfate with characteristic sulfur and oxygen isotope signatures. Elemental sulfur and tetrathionate are not only intermediate-valence state sulfur compounds that play a central role in sulfur oxidation pathways, but also key factors in shaping these isotope patterns.

## Introduction

The oxidation of sulfide minerals in oxic or anoxic environments drives the formation, and subsequent oxidation or reduction of various sulfur compounds; these are often associated with the generation of protons, creating a serious global environmental problem known as acid mine drainage (AMD; Schippers et al., 1996; Ramírez et al., 2004; Gleisner et al., 2006; Balci et al., 2007, 2012). Since sulfur is found in valence states ranging from +6 (sulfate) to −2 (sulfide), its transformation operates via several complex redox reaction pathways, many of which are microbially-mediated (Ehrlich, 1982). Elemental sulfur (S°), and mixed-valence-state sulfur species—molecules that consist of more oxidized sulfonate (−SO_3_) and more reduced sulfane (−S) components—including thiosulfate, (S_2_${\text{O}}_{3}^{2-}$), and tetrathionate, (S_4_${\text{O}}_{6}^{2-}$), have been observed during the microbially-mediated oxidation of monosulfides (e.g., galena, sphalerite) and disulfide (e.g., pyrite) minerals by both oxygen and ferric iron (Schippers et al., 1996; Schippers and Sand, 1999; Balci et al., 2007, 2012). Some mixed-valence-state sulfur species are stable at corrosive conditions (pH < 3; Williamson and Rimstidt, 1993; Xu and Schoonen, 1995; Schippers et al., 1996; Druschel et al., 2003; Bernier and Warren, 2007). For example, in acidic ferric iron (Fe^3+^)-rich solutions, the kinetics of abiotic trithionate and tetrathionate oxidation are several orders of magnitude slower than the formation of these polythionates from thiosulfate (Druschel et al., 2003).

The relative stability of these mixed-valence-state sulfur species in conditions found in acid-mine drainage raises the question of whether the production and oxidation state of different sulfur species is sulfide mineral–specific and how the fate of these mixed-valence-state sulfur species contributes to the overall corrosive conditions found in AMD (Druschel et al., 2003). For example, in contrast to the direct oxidation of pyrite, which lowers pH and contributes to the environmental acidity, the formation of aqueous hydrogen sulfide and its subsequent oxidation to elemental sulfur in acid conditions and in the presence of monosulfide minerals (e.g., sphalerite) consumes protons and may ameliorate acidic conditions (Schippers, 2004). Therefore, a variety of sulfur compounds may be important players in the overall redox reactions of sulfur in acid-mine drainage (Chambers and Trudinger, 1979; Steudel et al., 1987; Kelly, 1999; Schippers and Sand, 1999; Suzuki, 1999; Takano et al., 2000; Xu et al., 2000). Currently, our knowledge is incomplete regarding whether these sulfur species play a significant role in sulfur cycling since their oxidation involves multiple pathways and mixed-valence state species which have been difficult to quantify (Williamson and Rimstidt, 1993; Xu and Schoonen, 1995; Schippers et al., 1996; Schippers and Sand, 1999; Druschel et al., 2003).

A complicating factor is that microbial enzymatic reactions are faster than the kinetics of the abiotic reactions (Pronk et al., 1990; Hallberg et al., 1996; Friedrich et al., 2001), which not only leads to accelerated transformation of various sulfur compounds, but may also yield reaction products that differ from abiotic processes. The interplay between abiotic and microbially-catalyzed reactions plays a critical role in the biogeochemical sulfur cycle AMD conditions. However, particularly in acidic conditions it is difficult to distinguish reactions with mixed-valence state sulfur compounds carried out by microorganisms from purely abiotic reactions (Suzuki, 1974, 1999). Most of our knowledge about microbially-mediated sulfur redox chemistry in acidic conditions comes from studies carried out with *Acidithiobacillus ferrooxidans*, an acidophilic chemolithotrophic ferrous iron (Fe^2+^) and elemental sulfur oxidizer (Hazeu et al., 1986, 1988; Suzuki et al., 1994; Schippers et al., 1996; Gleisner et al., 2006; Balci et al., 2007, 2012; Thurston et al., 2010). Although, most studies have been done with *A. ferrooxidans*, phylogenetically similar organisms also perform a number of different sulfur redox reactions involving intermediate and mixed-valence state sulfur species (Bernier and Warren, 2007; Poser et al., 2014). *Acidithiobacillus thiooxidans* is another common elemental sulfur oxidizer commonly found in acidic environments (Knickerbocker et al., 2000; Smith et al., 2012). In contrast to *A. ferrooxidans, A. thiooxidans* is not able to oxidize ferrous iron to ferric iron. In experimental studies, this difference can be advantageous, because the sulfur transformations catalyzed by *A. thiooxidans* are not overprinted by concomitant reactions of sulfur species with ferric iron. Oxidation of mixed-valence state sulfur compounds including thiosulfate and tetrathionate by *Acidithiobacillus* spp. suggests that the rate of acid generation and the type and concentration of mixed-valence state sulfur species produced were specific to both the substrate and microbial species (Bernier and Warren, 2005, 2007).

Since sulfur oxidation, reduction, and disproportionation reactions are often accompanied by sulfur and oxygen isotope fractionation, the sulfur and oxygen isotope composition of sulfate, may provide insight into the oxidation pathways of these sulfur compounds. A wide range of sulfur isotope fractionation has been reported under various experimental conditions during the microbial oxidation of various sulfur compounds. For example, phototrophic oxidation of sulfide to elemental sulfur and elemental sulfur to sulfate produced no sulfur isotope fractionation (Fry et al., 1984, 1988; Kelly, 2008); a similar lack of sulfur isotope fractionation was reported for phototrophic oxidation of sulfite and thiosulfate to sulfate (Fry et al., 1985) and chemotrophic oxidation of thiosulfate to sulfate (Fry et al., 1986). On the other hand, significant sulfur isotope fractionation during chemotrophic oxidation of sulfide to sulfate (−10.5 to −18.0‰, normal isotope effect) and of sulfide to polythionates (+0.6 to +19‰, inverse isotope effect) has also been shown (Kaplan and Rittenberg, 1964). Fry et al. (1988) reported a normal sulfur isotope fractionation of −5.2‰ during abiotic oxidation of sodium sulfide (Na_2_S) by dissolved atmospheric oxygen (O_2_) in aqueous solution at pH 11 such that the product sulfate has a lighter δ^34^S_SO4_ signature than the sulfide from which it derives, which is in contrast to the inverse effect (reaction product is enriched in heavy isotope relative to reactant) associated with anaerobic oxidation of sulfide by photosynthetic bacteria. Apparent inverse (and normal) isotope effects can be caused by isotope exchange between co-existing species, such as between bisulfide (HS^−^) and hydrogen sulfide (H_2_S), between sulfur compounds with different redox state, such as sulfite and sulfide, or within a compound that has mixed valence state sulfur atoms, such as between sulfonate and sulfane in thiosulfate. This suggests that sulfur isotope fractionation is possibly caused by the formation of mixed-valence state sulfur species—even abiotically (Kaplan and Rittenberg, 1964; Goldhaber, 1983). Generally, relatively little is known about sulfur isotope fractionation during the oxidation of polythionates (Fry et al., 1986; Kelly, 2008; Alam et al., 2013).

Oxygen isotope fractionation during oxidation of sulfur compounds can be even more intricate than the sulfur isotope fractionation, because in addition to kinetic isotope fractionation, it involves the incorporation of oxygen from different sources, particularly from water and O_2_, and because some sulfur compounds, such as sulfite, rapidly exchange oxygen isotopes with water (e.g., Müller et al., 2013a,b; Wankel et al., 2014). For the best documented AMD process, pyrite oxidation, oxygen incorporation from O_2_ into sulfate ranges from 0 to 36% (Taylor et al., 1984; Balci et al., 2007; Tichomirowa and Junghans, 2009), with an associated kinetic isotope fractionation with respect to O_2_ (ε^18^O_SO4−O2_) between −11.4 to −4.3‰ (Taylor et al., 1984; Balci et al., 2007; Tichomirowa and Junghans, 2009), and an associated kinetic isotope fractionation with respect to water (ε^18^O_SO4−H2O_) between −4.4 to 4‰ (Table 1). The δ^34^S_SO4_ and δ^18^O_SO4_ thus hold potential as tracers for the oxidation pathways and microbial mediation of oxidative sulfur cycling. Furthermore, thanks to the incorporation of oxygen from O_2_ during sulfur oxidation, the oxygen isotope signature of sulfate may help decipher the geologic history of atmospheric oxygen (Bao, 2015).

**Table 1:** Compilation of previous relevant studies.

**Experimental condition**	**H_2_O (%)**	**ε^18^O_SO4−H2O_ (‰)**	**ε^18^O_SO4−O2_ (‰)**	**ε^34^S_SO4−S0_ (‰)**	**ε^34^S_S0−S2_ (‰)**	**ε^34^S_SO4−metalSulfide_ (‰)**	**ε^34^S_SO4/SxOy−S2/SxOy_ (‰)**	**References**
Oxidation of H_2_S to S^o^ by *A. thiooxidans*					−1.2 to +2.5	n.a		Kaplan and Rittenberg, 1964
Phototrophic oxidation of S^2−^ to S^o^ by *Chromatium* sp.					−3.6 to −10			Kaplan and Rittenberg, 1964
Phototrophic oxidation of S^2−^ to ${\text{SO}}_{4}^{2-}$ by *Chromatium* sp.							+0.9 to −2.9	Kaplan and Rittenberg, 1964
Phototrophic oxidation of S^2−^ to S_X_${\text{O}}_{6}^{2-}$ by *Chromatium* sp.							+4.9 to +11.2	Kaplan and Rittenberg, 1964
Oxidation of S^2−^ to ${\text{SO}}_{4}^{2-}$ ^(a)^ by *A. thiooxidans*							−18 to −10.5	Kaplan and Rittenberg, 1964
Oxidation of S^2−^ to S_*x*_O_6_ ^(a)^ by *A. thiooxidans*							+0.6 to +19	Kaplan and Rittenberg, 1964
Aerobic S^o^ oxidation with *T. Concretivorus*				−0.1 to +1.4				Kaplan and Rittenberg, 1964
Aerobic S^o^ (soil slurry)	100	0		<+2.3				Mizutani and Rafter, 1969
Pyrite oxidation with *A. ferrooxidans*	70	+3.5	−11.4					Taylor et al., 1984
Abiotic pyrite oxidation	n.d	n.d	−4.3					Taylor et al., 1984
Oxidation of S_2_${\text{O}}_{4}^{2-}$ to ${\text{SO}}_{4}^{2-}$ by *Paracoccus versutus*	n.d	n.d	n.d				+0.4	Fry et al., 1986
Oxidation of S_2_${\text{O}}_{3}^{2-}$ to ${\text{SO}}_{4}^{2-}$by *Halothiobacillus neapolitanus*	n.d	n.d	n.d				+1.2 to +2.9	Kelly, 2008
Aerobic pyrite oxidation with *A. ferrooxidans*	85 to 92	−4.0 to +4.0	−10 to −11					Balci et al., 2007
Anaerobic pyrite oxidation by Fe^3+^(aq) with/without by *A. ferrooxidans*	94 to 95	+3.6						Balci et al., 2007
Anaerobic pyrite oxidation	87 to 97	+3.3 to +4.0						Heidel et al., 2009
Abiotic/Submerged pyrite oxidation	72 to 85	n.d				−0.0 to +0.3		Tichomirowa and Junghans, 2009
Abiotic/Wet/Dry pyrite oxidation	64 to 81	n.d				−0.4 to +0.0		Tichomirowa and Junghans, 2009
Anoxygenic phototropic S oxidation with *Chlorobium tepidum*		n.d		1.9 ± 0.8				Zerkle et al., 2009
Abiotic pyrite oxidation	91	+4.1 to +4.8	−8.4	n.d				Heidel and Tichomirowa, 2010
Anaerobic chalcopyrite oxidation by Fe^3+^(aq) with/without by *A. ferrooxidans*	92 to 94	+3.8		n.d	−3.0	−3.8		Thurston et al., 2010
Aerobic chalcopyrite oxidation by *A. ferrooxidans*	92 to 95	+6.4		n.d		−1.5		Thurston et al., 2010
Abiotic oxidation of chalcopyrite O_2_(aq)	57	+5.2 to 7.4		n.d		−0.5		Thurston et al., 2010
Sphalerite oxidation by Fe(III)aq with/without *A. ferrooxidans*	96	+7.5 to +8.2		−2.8 to −2.9	+0.3 to +0.6	−2.6 to −2.4		Balci et al., 2012
Aerobic sphalerite oxidation with *A. ferrooxidans*	92 to 96	+9.5 to +8.1				0		Balci et al., 2012
Aerobic S° oxidation with *A. thiooxidans*	84 to 97	−6.2 to −0.9		0.4 ± 0.8				Smith et al., 2012
Aerobic S° oxidation by *A. ferrooxidans* (short term, 10 days)		n.d		−1.8				Balci et al., 2012
Aerobic S° oxidation by *A. ferrooxidans* (short term, 20 days)	100	8.3		−1.1				Balci et al., 2012
Aerobic oxidation of S_2_${\text{O}}_{3}^{2-}$ to ${\text{SO}}_{4}^{2-}$ by *Thiomicrospria crunogena*							−1.9 to + 4.6	Alam et al., 2013
Aerobic oxidation of S_2_${\text{O}}_{3}^{2-}$ to ${\text{SO}}_{4}^{2-}$ by *Tetrathiobacter kashmirensis*							−4.9 to −0.8	Alam et al., 2013
Aerobic oxidation of S_2_${\text{O}}_{3}^{2-}$ to ${\text{SO}}_{4}^{2-}$ by *Paracoccus pantothrophus*							−5.8 to 1.8	Alam et al., 2013
Aerobic oxidation of elemental sulfur by *A. thioxidans*	58 to 87	−4.2		−1.8 to 0				Current study
Aerobic oxidation of tetrathionate by *A. thioxidans*	60 to 62						+2.9 to +3.5	Current study

(a) *Determined as a minor product*.

Given the complexity of abiotic and microbially-catalyzed sulfur transformations, and the large number of potentially involved sulfur compounds, it becomes essential to identify what shapes the δ^34^S_SO4_ and δ^18^O_SO4_ of sulfate produced in acid-mine drainage. We identified two sulfur compounds with these attributes: tetrathionate and elemental sulfur. The choice of tetrathionate is based on the observation that in presence of ferric iron, which is common in acid-mine drainage, thiosulfate is quickly transformed into tetrathionate:

(1) $$\begin{array}{l} 2{\text{Fe}}^{3+}+2{\text{S}}_{2}{{\text{O}}_{3}}^{2-}\to 2{\text{Fe}}^{2+}+{\text{S}}_{4}{{\text{O}}_{6}}^{2-} \end{array}$$

Thiosulfate itself is the first reaction product in the oxidation of acid-insoluble metal sulfides such as pyrite, (FeS_2_), molybdenite (MoS_2_), or tungstenite (WS_2_), the so-called thiosulfate mechanism (Schippers and Sand, 1999), while tetrathionate is much more stable in AMD conditions. Microorganisms are likely to take advantage of the relative kinetic stability of tetrathionate by catalyzing the degradation of this compound. Moreover, the abiotic decomposition of tetrathionate produces sulfate and disulfane/monosulfonic acid (Schippers et al., 1996). The latter can react with other sulfur species to yield a suite of sulfur compounds with intermediate oxidation state, including elemental sulfur, thiosulfate, tri- and pentathionate (Schippers et al., 1996). From the perspective of isotope fractionation, tetrathionate may preserve the isotope signature of thiosulfate, whereas the conversion of tetrathionate into various other sulfur compounds offers the potential to express a variety of diagnostic isotope fractionations. If oxygen from O_2_ is incorporated during these processes, it may end up in the final oxidation product sulfate, leaving a unique isotopic fingerprint. Moreover, the expression of the oxygen and sulfur isotope fractionation in this process could strongly depend on the involved microorganisms, as well as acidity, and concentration of oxidants such as O_2_ and ferric iron. Elemental sulfur, the second chosen key component is not only a product of the decomposition of tetrathionate, but also central in the oxidation of acid-soluble metal sulfides, such as sphalerite (ZnS), where the first oxidation reaction product are polysulfides, which under acid conditions quickly decompose to form elemental sulfur (polysulfide mechanism; Schippers and Sand, 1999). As for tetrathionate, the abiotic oxidation of elemental sulfur is kinetically slow relative to the processes that form this compound, providing microorganisms with the opportunity to catalyze this process.

To date, there have been no studies exploring sulfur or oxygen isotope fractionation during microbial oxidation of tetrathionate to sulfate under acidic conditions (pH < 4) where the accumulation and oxidation of tetrathionate occurs and influences the sulfur cycle (Druschel et al., 2003) and there are no data on the sulfur or oxygen isotope fractionation during microbial oxidation of elemental sulfur by *A. thiooxidans* under AMD conditions. In this pilot study, we assess if tetrathionate indeed holds the potential to be a key compound in shaping the δ^34^S_SO4_ and δ^18^O_SO4_ and we determine the sulfur and oxygen isotope fractionation during microbial oxidation of elemental sulfur by *A. thiooxidans*.

## Materials and methods

### Bacterial strain, media, and growth conditions

The acidophilic sulfur oxidizing bacterium *A. thiooxidans* (11,478; formerly, *Thiobacillus thiooxidans*) was used for all the experiments and was obtained from Deutsche Sammlung von Mikroorganismen und Zellkulturen (DSMZ) culture collection. Before use in the experiments, the strain was grown chemolithoautotrophically in batch culture in a medium containing the modified basal salts solution supplemented with either tetrathionate (20 mM K_2_S_4_O_6_) or elemental sulfur (1 g/100 mL). The basal salts consist of 0.6 g/L NH_4_Cl, 0.2 g/L MgCl_2_.6H_2_O, 0.1 g/L K_2_HPO_4_; 0.10 g/L KCI; 0.01 g/L Ca(NO_3_)_2_. The medium was prepared by adding these salts to 1 L of deionized water and the pH of the medium was adjusted to 4 with trace-metal grade HCl. Then the culture medium was autoclaved for 25 min for the experiments with elemental sulfur and, to prevent oxidation, filter sterilized for the experiments with tetrathionate. Bacteria were harvested in late exponential phase (at an optical density at 440 nm of between 0.27 and 0.28), centrifuged, and re-suspended in the medium and used in the biological oxidation experiments for 2 h. For the cultures grown on elemental sulfur, sequential centrifugation was applied to obtain sulfur-free cells: first the cultures were centrifuged at 1,800 rpm to remove elemental sulfur particles. Subsequently, the supernatant containing cells was further centrifuged at 10,000 rpm for 10 min. The cell pellet was then re-suspended in 5 ml medium and used for elemental sulfur oxidation experiments. The cell densities in the biological experiments were determined by phase contrast microscopy: 10^7^ cell/ml (with S° as a substrate) or 10^6^ cell/ml (with tetrathionate). *A. thiooxidans* was subcultured three times before being used in the biological experiments.

### Biological and abiotic oxidation of S° and S_4_${\text{O}}_{6}^{2-}$ under aerobic conditions

A sulfate-free medium was used as an experimental solution with the same basal salt concentration as above to ensure that the sulfate recovered after chemolithotrophic growth was exclusively produced from the oxidation of elemental sulfur or tetrathionate. Two hundred and fifty milliliters of microbiological medium was placed into 500 ml Erlenmeyer flasks and autoclaved at 121°C for 20 min. The flasks were then kept in a sterile hood under UV light for 25 min to decontaminate their surfaces. Prior to use, the elemental sulfur was ground and sieved to a grain size of <63 μm. For sterilization, elemental sulfur was soaked with 70% ethanol and spread in a thin even layer under UV radiation (germicidal) in a sterile hood for 30 min. Then the elemental sulfur was immediately placed in sterile experimental containers. To initiate the experiments, 2.5 g of elemental sulfur and 5 ml (5 × 10^7^ cell) of the *A. thiooxidans* cell suspension were added to each flask and subsequently adjusted to a pH of 4 with trace metal grade HCl. For the experiments with tetrathionate, 250 ml of medium containing 20 mM K_2_S_4_O_6_ was placed into 500 ml Erlenmeyer flask and pH of the medium was adjusted to 4 with HCI at 25°C and filter-sterilized. The flasks used for elemental sulfur and tetrathionate oxidation experiments were loosely sealed with a sterilized thin aluminum film such that gas exchange between the headspace and ambient air could take place. The biological incubations were done with waters with different δ^18^O values (−4.4‰; referred to as W1), +58.0‰ (W2) and +84.4‰ (W3). For each δ^18^O of the water, 15 flasks were used. This experimental design serves to determine the relative contribution of water to the oxygen in the produced sulfate. All the biological experiments were incubated at 25°C for 30 days with continuous shaking (150 rpm) in a room where temperature and humidity were held constant. One abiotic-control experiment with a δ^18^O of water of −4.4‰ was conducted with elemental sulfur, and two abiotic control experiments with different δ^18^O of water (−4.4‰ and +84.4‰) were conducted with tetrathionate.

### Sampling for aqueous sulfur species and analysis

Each flask from the experiments with different δ^18^O of water was terminated at various time points and pH was directly measured in the flask with a pH meter (WTW model 340i) following a 4-point calibration using pH 1.0, 2.0, 4.0, and 7.0 buffers. A 5 ml of aliquot was collected at each time point and immediately filtered through a 0.2 μm syringe filter into a sterile 5 ml dark tube in the anaerobic chamber (Coylab) and kept at −20°C for determination of the concentrations of sulfur species including thiosulfate and sulfite. Another 5 ml aliquot was collected at each time point and was used for elemental sulfur quantification. Elemental sulfur was quantified by extraction with acetone/water (19:1) followed by the cyanolysis methods (Kelly and Wood, 1994, 1998; Beard et al., 2011). Thiosulfate concentrations were determined by applying the decoloration of methylene-blue method at 670 nm in acidic conditions (Kletzin, 1989). The bimane derivatization method was used to quantify sulfite concentrations (Zopfi et al., 2004). A cold-cyanolysis protocol was used to determine tetrathionate concentrations at the end of the incubation (Kelly et al., 1969). An additional 5 ml aliquot filtered through 0.2 μm syringe filter was analyzed for sulfate concentrations by ion exchange chromatography using a Dionex ion chromatograph. The following mass balance can be used to estimate the percentage of tetrathionate consumed (see Table 2 for sulfur concentrations of the individual compounds):

(2) $$\begin{array}{l} {\left[{\text{S}}_{4}{\text{O}}_{6}\right]}_{\text{consumed}}={\left[{\text{S}}_{4}{\text{O}}_{6}\right]}_{\text{initial}}-(4^{*}{\left[{\text{S}}_{4}{\text{O}}_{6}\right]}_{\text{final}}+{\left[{\text{SO}}_{4}\right]}_{\text{final}}\\ \;+2^{*}{\left[{\text{S}}_{2}{\text{O}}_{3}\right]}_{\text{final}}+{\left[{\text{SO}}_{3}\right]}_{\text{final}}+{\left[{\text{S}}^{0}\right]}_{\text{final}})/4 \end{array}$$

It must be noted that the calculated value for [S_4_O_6_]_consumed_ represents a minimum estimate, as sulfur may be accumulated in the form intermediate-valence state species that are not measured, or lost from the experiment in gaseous form, such as SO_2_.

**Table 2:** Changes in solution chemistry during microbial oxidation of elemental sulfur.

**Time (hours)**	**W1 Experiments**				**W2 Experiments**				**W3 Experiments**			
	**pH**	**SO_4_ (mM)**	**S_2_O_3_ (mM)**	**SO_3_ (μM)**	**pH**	**SO_4_ (mM)**	**S_2_O_3_ (mM)**	**SO_3_ (μM)**	**pH**	**SO_4_ (mM)**	**S_2_O_3_ (mM)**	**SO_3_ (μM)**
0	3.15	0.02	0.0	n.d	3.09	0.02	0	n.d	3.15	0.02	n.d	n.d
4	3.09	0.02	0.0	n.d	3.01	0.04	0.000	n.d	3.08	0.02	0.000	n.d
24	2.60	5.7	0.029	n.d	2.81	3.5	0.024	n.d	2.81	4.1	0.022	n.d
33	2.55	10.5	0.048	0.05	2.52	14.2	0.051	n.d	2.55	9.6	0.040	n.d
48	2.20	16.2	0.107	n.d	2.39	15.3	0.073	n.d	2.28	13.4	0.073	n.d
60	2.09	23.5	0.099	n.d	2.17	16.3	0.086	n.d	1.99	26.3	0.016	n.d
72	1.70	38.4	0.063	n.d	1.89	29.1	0.095	n.d	1.74	31.2	0.024	n.d
84	1.42	49.3	n.d	n.d	1.71	28.4	n.d	n.d	1.56	36.3	n.d	n.d
96	1.52	52.6	0.007	0.24	1.68	48.7	0.001	0.16	1.5	51.2	0.005	0.14
124	1.42	63.2	n.d	n.d	1.52	52.3	n.d	n.d	1.38	58.6	n.d	n.d
146	1.31	68.3	n.d	n.d	1.47	64.3	n.d	n.d	1.3	62.7	n.d	n.d
168	1.26	76.2	n.d	n.d	1.34	n.d	n.d	n.d	1.18	96.4	n.d	n.d
264	0.83	131.3	n.d	n.d	1.09	98.4	n.d	n.d	0.81	115.2	n.d	n.d
336	0.80	133.2	0.001	n.d	1.13	101.63	n.d	n.d	0.77	151.7	n.d	n.d
504	0.74	155.7	n.d	n.d	0.92	120.33	n.d	n.d	0.72	148.6	n.d	n.d
720	0.72	158.2	0.001	n.d	0.74	154.98	0.001	n.d	0.68	161.6	n.d	n.d

*n.d., not determined*.

### Isotopic analyses

Filtered samples (10 ml) from each experimental time point were kept tightly sealed and frozen until the δ^18^O of water could be analyzed. The remaining reaction medium (~200 ml) was filtered through a 0.2 μm Millipore filter and brought to a pH 3 using dilute NaOH. A 10% (wt/wt) BaCI_2_ solution was added and the product barium sulfate (BaSO_4_) allowed to settle overnight. The BaSO_4_ precipitate was filtered and collected on a 0.2 μm Millipore filter, washed first with 100 ml of 1N HCl, then rinsed 3 times with a total of 500 ml of deionized water. The BaSO_4_ samples were then dried. The sulfur isotope analysis of initial tetrathionate and elemental sulfur was performed without additional treatment on K_2_S_4_O_6_ and S^0^, respectively. No oxygen isotope analysis of K_2_S_4_O_6_ was performed.

Sulfur and oxygen isotope ratios were determined by continuous flow isotope ratio mass spectrometry (CF-IRMS) using an elemental analyzer (for sulfur isotopes—at the Max Plank Institute for Marine Microbiology, Bremen) or a Thermo-Finnigan TC/EA at 1,450°C (for oxygen isotopes—in the Godwin Laboratory at the University of Cambridge). The oxygen and sulfur isotope composition is expressed relative to the Vienna Standard Mean Ocean Water (V-SMOW), and Vienna Canyon Diablo Troilite (V-CDT) standards, respectively, using the standard delta notation:

(3) $$\begin{array}{l} \delta^{18}\text{O}\;\text{or}\;\delta^{34}\text{S}={\left({\text{R}}_{\text{sample}}/{\text{R}}_{\text{standard}}-1\right)}^{*}1000‰, \end{array}$$

where R is the ratio of the heavy to light isotope (^18^O/^16^O or ^34^S/^32^S) of sample and reference, respectively. For sulfur isotope measurements, IAEA S1 (−0.3‰), S_2_ (+21.7‰), SO-5 (+0.49‰), and SO-6 (−34.05‰) were analyzed for calibration and normalization purposes, the error reported on the analyses is based on replicate analysis of these standards within each run and was generally better than 0.2‰. Oxygen isotope ratios of sulfate were normalized to NBS 127 (δ^18^O = +8.6‰) which was run at the beginning and end of each block of five samples. Samples for oxygen isotopes in sulfate were run in triplicate and the average and standard deviation of these triplicate analyses is reported (generally better than 0.5‰).

The δ^18^O of water was determined by analyzing CO_2_ gas that had equilibrated with 200 μl aliquots at 40°C in septum-capped vials. Raw data were corrected for the H_2_O–CO_2_ oxygen isotope fractionation, and then adjusted for small instrumental effects using results obtained for water standards that had been previously calibrated against VSMOW and SLAP. Replicate analyses agreed within less than ±0.1‰. The δ^18^O of O_2_ in the flasks was analyzed through injection into the TC/EA and the δ^18^O value of O_2_ was +23.5 ± 0.5‰ (*n* = 4) at the beginning of study and +24.3 ± 1.7‰ at the end of the experiments (30 days).

### Calculation of the source of oxygen and isotopic fractionation during elemental sulfur oxidation

The δ^18^O_SO4_ of sulfate produced during elemental sulfur oxidation depends on: (1) the fraction of oxygen atoms coming from water (X), (2) the δ^18^O of the water, (3) any oxygen isotope fractionation between water oxygen and sulfate oxygen during the incorporation of oxygen atoms from water during oxidation (ε^18^O_SO4−H2O_), (4) the fraction of oxygen atoms derived from molecular O_2_ (1−X), (5) the δ^18^O of atmospheric oxygen, and (6) any oxygen isotope fractionation between atmospheric oxygen and sulfate oxygen during the incorporation of oxygen atoms from atmospheric oxygen during oxidation (ε^18^O_SO4−O2_) (Mandernack et al., 1995; Balci et al., 2007; Thurston et al., 2010). The overall contributions of these factors is given in the following equation:

(4) $$\begin{array}{l} \delta^{18}{\text{O}}_{\text{SO}4}={\text{X}}^{*}\left(\delta^{18}{\text{O}}_{\text{H}2\text{O}}+\varepsilon^{18}{\text{O}}_{\text{SO}4-\text{H}2\text{O}}\right)\\ +\;{\left(1-\text{X}\right)}^{*}\left(\delta^{18}{\text{O}}_{\text{O}2}+\varepsilon^{18}{\text{O}}_{\text{SO}4-\text{O}2}\right) \end{array}$$

which can be simplified and rearranged to form:

(5) $$\begin{array}{l} \delta^{18}{\text{O}}_{\text{SO}4}={\text{X}}^{*}(\delta^{18}{\text{O}}_{\text{H}2\text{O}}+\varepsilon^{18}{\text{O}}_{\text{SO}4-\text{H}2\text{O}}-\delta^{18}{\text{O}}_{\text{O}2}\\ \;-\varepsilon^{18}{\text{O}}_{\text{SO}4-\text{O}2})+\left(\delta^{18}{\text{O}}_{\text{O}2}+\varepsilon^{18}{\text{O}}_{\text{SO}4-\text{O}2}\right) \end{array}$$

Because the δ^18^O of atmospheric oxygen is constant and the experimental conditions are performed such that ε_SO4−H2O_ and ε_SO4−O2_ are expected to be constant, the fraction of oxygen derived from water can be determined by replicate experiments with variable δ^18^O for water. A linear least squares regression for δ^18^O_SO4_ vs. δ^18^O_H2O_ has a slope, X, equal to the fraction of oxygen derived from water, and (1 − X) is the remaining fraction from O_2_. The average δ^18^O_H2O_ from the initial and final time points were used for these graphs.

Equation (5) has two unknowns, the oxygen isotope fractionation for the incorporation of water into sulfate (ε^18^O_SO4−H2O_) and the oxygen isotope fractionation for the incorporation of atmospheric oxygen into sulfate (ε^18^O_SO4−O2_). If the produced sulfate is entirely derived from oxygen-atoms from water (slope, X = 1 or close to 1) equation 5 is simplified and ε^18^O_SO4−H2O_ can be estimated from the intercept with the y-axis:

(6) $$\begin{array}{l} \delta^{18}{\text{O}}_{\text{SO}4}=\delta^{18}{\text{O}}_{\text{H}2\text{O}}+\varepsilon^{18}{\text{O}}_{\text{SO}4-\text{H}2\text{O}} \end{array}$$

### Calculation of contribution of oxygen from water during tetrathionate oxidation

In the case of tetrathionate oxidation, the δ^18^O_SO4_ of sulfate generated depends also on the oxygen isotope composition of tetrathionate. If all oxygen from tetrathionate ends up in sulfate, i.e., no oxygen from tetrathionate is lost to water during the oxidation, the contribution of tetrathionate-oxygen to sulfate-oxygen can be assessed from the following chemical reaction:

(7) $$\begin{array}{l} {\text{S}}_{4}{{\text{O}}_{6}}^{2-}+3.5{\text{O}}_{2}+3{\text{H}}_{2}\text{O}\to 4\text{S}{{\text{O}}_{4}}^{2-}+6{\text{H}}^{+} \end{array}$$

This indicates that as much as 37.5% of oxygen in the resultant sulfate could be derived from the initial tetrathionate, 43.75% from O_2_, and as little as 18.75% from water. Analogous to Equation (5), the approach with using water with different δ^18^O allows us to determine the actual contribution of oxygen from water, however, does not permit us to differentiate between the contribution of oxygen from O_2_ and tetrathionate. To achieve the latter, additional experiments would need to be carried out in which different δ^18^O of O_2_ are used.

### Estimates of error on measured δ^18^O_SO4_ values from contamination with sulfate

In our biological sulfur oxidation experiments, a relatively large background concentration of sulfate was measured during the first 24 h after inoculation. This initial concentration of sulfate may be due to analytical error in the ion chromatograph or due to sulfate carried over with the inoculum. The impact on the δ^18^O_SO4_ at the end of the experiments is low as this sulfate is insignificant relative to the total sulfate produced during the experiment. However, this initial sulfate corresponds to as much as 60% of the total sulfate in the first 24 h of the incubation. To determine the δ^18^O_SO4_ of the produced sulfate in the early stage of oxidation a correction for the initially present sulfate in the culture medium was applied. We used the mass balance equation

(8) $$\begin{array}{l} \delta^{18}{\text{O}}_{\text{SO}4\text{\_measured}}={\text{X}}^{*}\left(\delta^{18}{\text{O}}_{\text{SO}4\text{\_initial}}\right)\\ +\;{\left(1-\text{X}\right)}^{*}\left(\delta^{18}{\text{O}}_{\text{SO}4\text{\_product}}\right), \end{array}$$

where X is the percentage of sulfate derived from the initial sulfate; δ^18^O_SO4_initial_ corresponds to the δ^18^O of initial sulfate; (1 − X) refers to the percentage of sulfate derived from biological oxidation of elemental sulfur at a given point, and δ^18^O_SO4_product_ and δ^18^O_SO4_measured_ are the δ^18^O of the measured sulfate in solution, and δ^18^O of the produced sulfate at a given time point.

## Results

### Changes in solution chemistry during elemental sulfur oxidation

**Figure 1:**
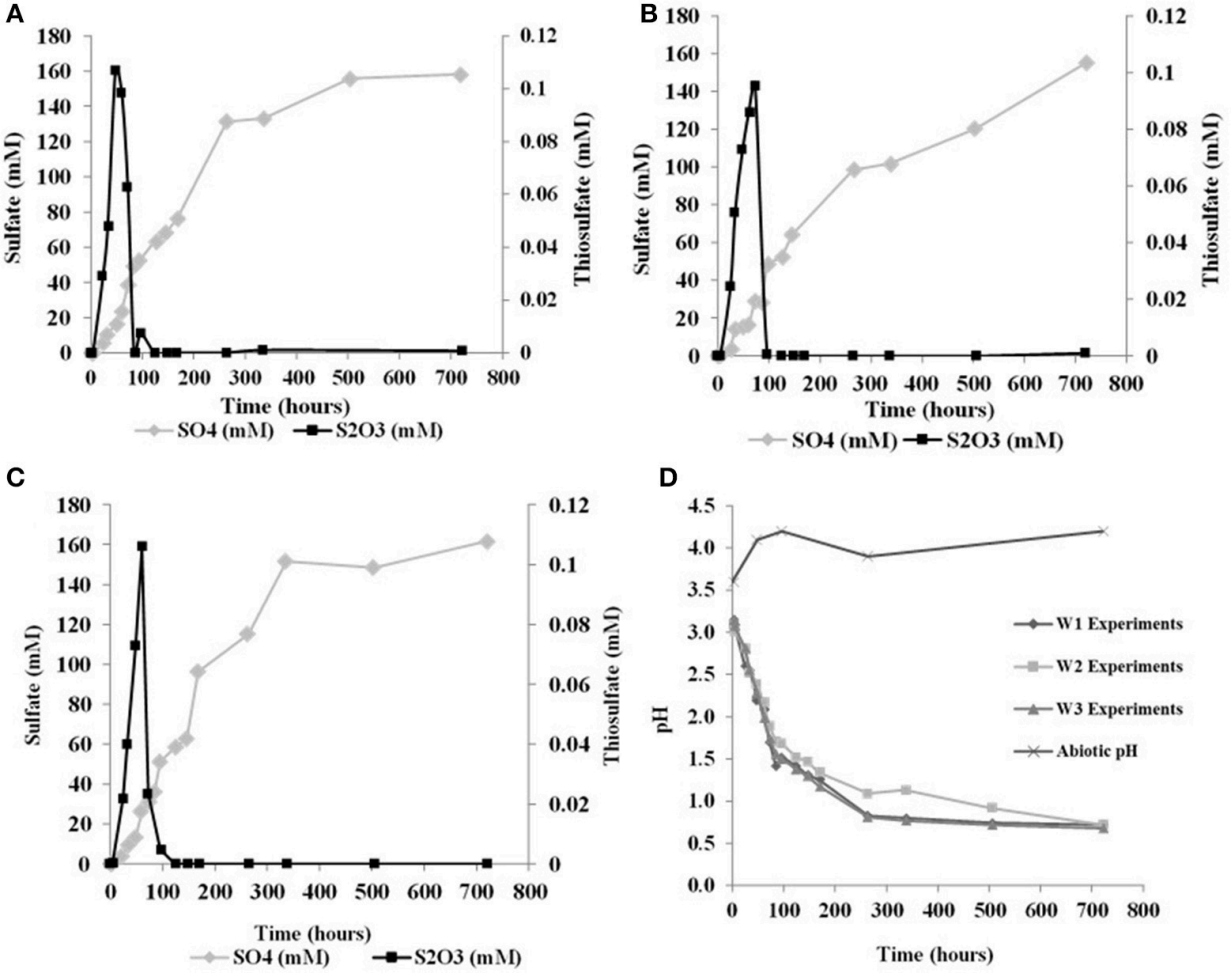
Changes in solution chemistry during microbial oxidation of elemental sulfur **(A)**, W1 Experiments (δ^18^O_H2O_ −5.5‰) **(B)** W2 Experiments (δ^18^O_H2O_ +58.4‰) **(C)** W3 Experiments (δ^18^O_H2O_ +84.4‰) **(D)** changes in pH vs. time.

The initial sulfate concentration in all the biological experiments was ~20 μM. In all experiments, an initial lag phase delayed sulfate production (Table 2, Figure 1). An initial lag phase is commonly observed during batch culture experiments, particularly during elemental sulfur oxidation (Yu et al., 2001; Balci et al., 2012; Smith et al., 2012). The length of this lag phase may depend on the pH of the medium, substrate availability, chemical composition of the experimental vs. culture-growing medium in addition to the inoculation stage of culture (Yu et al., 2001; Swinnen et al., 2004). Since a direct contact between *A. thiooxidans* and sulfur grains is required to overcome the hydrophobic nature of elemental sulfur and to initiate the process of microbial oxidation, it may be required for bacteria to attach to the surface of elemental sulfur; this further contributes to a longer lag phase (Knickerbocker et al., 2000; Yu et al., 2001). The initial lag phase is characterized by a slow increase in sulfate concentrations and high rates production of thiosulfate (Figure 1). A small decrease in pH (to 2.8) occurred in each of the biological experiments during this initial lag phase. Following this lag phase, sulfate concentrations increased with decreasing thiosulfate concentration over the course of the experiment (Figure 1). In our experiments, the rate of pH change slowed when the pH reached 1.0 with no further changes after the pH of the solution measured at 0.7. In the abiotic control, the pH of experiment rose after 48 h from 3.6 to 4.1 and remained invariant at 4.2 over the experiment (Figure 1D). These abiotic control experiments produced minimal sulfate (7.2 μM), indicating that >95% of sulfate produced in the biotic experiments resulted from microbial activity.

Sulfite was only found in a few samples over the course of each experiment, indicating sulfite was rapidly transformed into other sulfur species (Table 2). Prior studies also have reported the formation of some mixed-valence-state sulfur species, like thiosulfate, during the oxidation of elemental sulfur (Rohwerder and Sand, 2003). In the abiotic control experiments, thiosulfate and sulfite were not found over the course of the experiments.

### Changes in solution chemistry during tetrathionate oxidation

**Figure 2:**
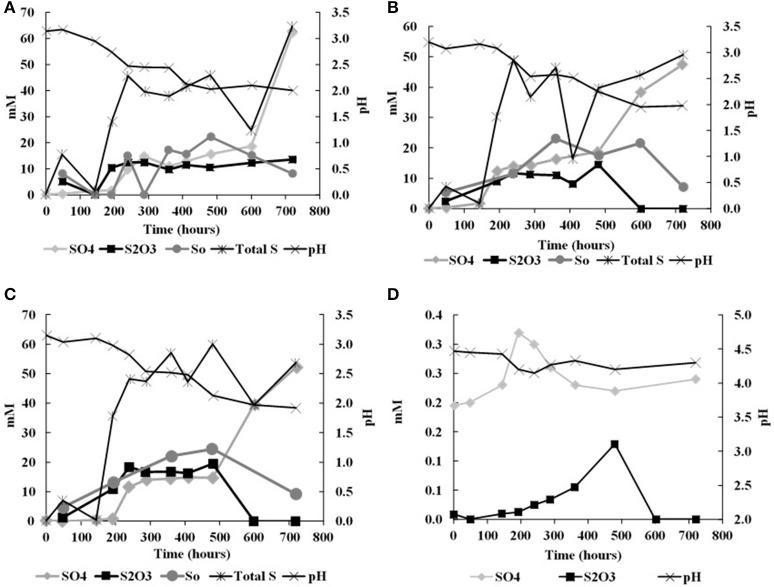
Changes in solution chemistry during microbial oxidation of tetrathionate **(A)**, W1 Experiments (δ^18^O_H2O_ −5.5‰) **(B)** W2 Experiments (δ^18^O_H2O_ +58.4‰) **(C)** W3 Experiments (δ^18^O_H2O_ +84.4‰) **(D)** abitoic oxidation of tetrathionate.

In the biological experiments with *A. thiooxidans* grown on tetrathionate (initial concentration 20 mM, corresponding to 80 mM sulfur), sulfate, thiosulfate and elemental sulfur were produced (Figures 2A–C, Table 3). At the end of the month-long incubation 78% of the initial tetrathionate was oxidized to sulfate (62.4 mM), 2.9% of the sulfur from tetrathionate was found in the form of elemental sulfur (2.3 mM), and 4.1% remained as tetrathionate (0.82 mM) for W1. For W2, 59% of the tetrathionate was oxidized to sulfate (47.5 mM), 4% was converted to elemental sulfur (3.2 mM), and 2.7% remained as tetrathionate (0.53 mM). For W3, 65% of the tetrathionate was oxidized to sulfate (52.1 mM), 2% was converted to 3.2 mM elemental sulfur (1.6 mM), and 3.6% remained as tetrathionate (0.72 mM). These findings mean that in all experiments, a substantial proportion of the sulfur supplied in the form of tetrathionate remain unaccounted for. This “missing sulfur” corresponds to 15% (12.02 mM), 34% (27.18 mM), and 29% (23.42 mM) for W1, W2 and W3, respectively.

**Table 3:** Changes in solution chemistry during microbial oxidation of tetrathionate.

**Time (hours)**	**W1 Experiments**				**W2 Experiments**				**W3 Experiments**			
	**pH**	**SO_4_ (mM)**	**S_2_O_3_ (mM)**	**S° (mM)**	**pH**	**SO_4_ (mM)**	**S_2_O_3_ (mM)**	**S° (mM)**	**pH**	**SO_4_ (mM)**	**S_2_O_3_ (mM)**	**S° (mM)**
0	3.14	0.21	n.d	n.d	3.2	0.16	n.d	n.d	3.15	0.20	n.d	n.d
48	3.17	0.38	5.20	4.70	3.07	0.38	2.35	2.2	3.04	0.00	1.20	4.60
144	2.95	1.60	n.d	n.d	3.16	1.67	n.d	n.d	3.10	0.42	n.d	n.d
192	2.74	7.50	10.29	n.d	3.08	12.41	8.94	n.d	2.98	0.81	10.87	13.10
240	2.47	9.70	12.44	11.00	2.85	14.02	11.71	11.50	2.82	11.60	18.31	n.d
288	2.45	14.80	12.44	n.d	2.54	14.22	11.28	n.d	2.54	14.00	16.68	n.d
360	2.44	11.07	9.80	7.20	2.57	16.27	11.05	8.10	2.52	14.34	16.74	9.2
408	2.13	12.76	11.53	5.60	2.52	n.d	8.20	n.d	2.48	14.77	16.21	n.d
480	2.03	15.63	10.50	9.30	2.25	18.80	6.72	7.50	2.13	14.72	19.43	6.4
600	2.0	18.57	n.d	6.20	1.95	38.40	n.d	5.60	1.97	39.40	0.00	n.d
720	2.00	62.40	n.d	2.30	1.98	47.50	n.d	3.20	1.92	52.10	0.00	1.6

*n.d., not determined*.

During each biological experiment, the pH dropped from 3.2 to 2.0 by the end of the experiments. The production of thiosulfate decreased with higher generation of acid and the lowest concentration of thiosulfate accompanied the highest concentration of sulfate in all the experiments (Figures 2A–C). We found that elemental sulfur formed in the experiments, and its concentration decreased with increasing incubation time (Figure 2, Table 3). The rate of sulfate, thiosulfate and elemental sulfur production differed slightly among the experiments. The abiotic control experiments run with tetrathionate as a substrate did not produce significant amounts of sulfate or thiosulfate over the course of the experiments (0.24 and 0.13 mM, respectively) and remained at nearly constant pH (Figure 2D). Neither elemental sulfur nor sulfite was found in these abiotic experiments.

### Oxygen and sulfur isotopic composition of sulfate in experiments with elemental sulfur

**Figure 3:**
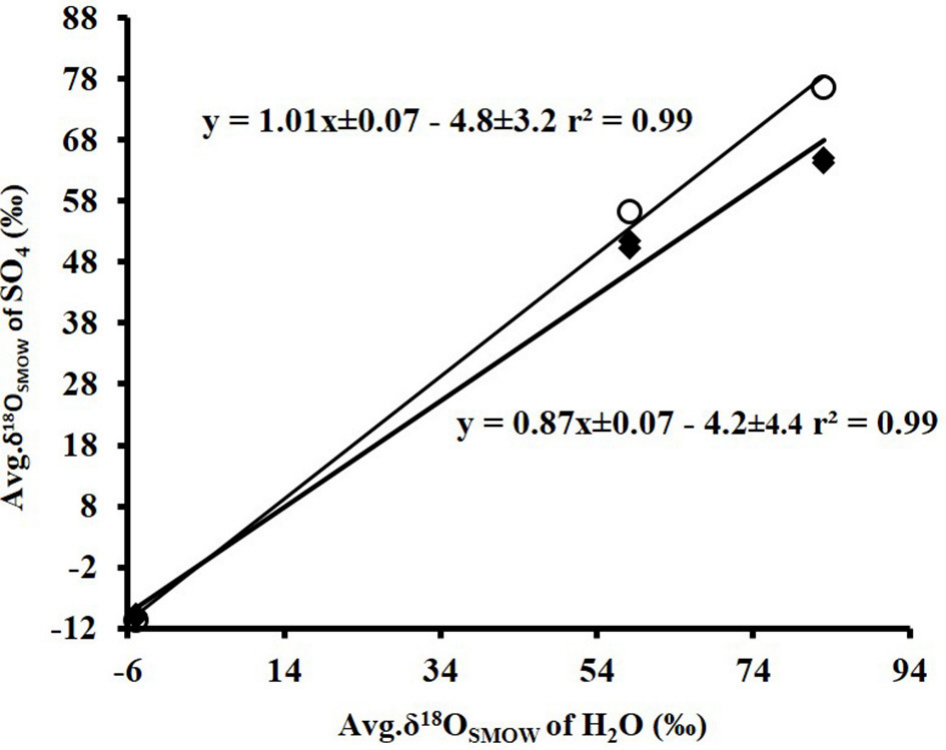
Plot of the average δ^18^O_SO4_ (*n* = 37)(•) produced from microbial oxidation of elemental sulfur and (◦) produced from main stage oxidation vs. average δ^18^O_H2O_ (*n* = 3) values and used in the experiments (see Table 1).

A cross plot of δ^18^O_SO4_ vs. δ^18^O_H2O_ for the initial (lag phase) stage of the experiment shows a strong correlation (*r*^2^ > 0.99). The slope (X) of this linear regression was used to estimate the contribution of water-derived oxygen into the product sulfate. During the prolonged lag phase where we observed low sulfate production coupled with high thiosulfate concentrations, the slope indicates that between 58 and 66% of the sulfate-oxygen was derived from water, with the remaining 34–42% derived from atmospheric oxygen (Table 4). During the main stage of the experiments (high sulfate production and low thiosulfate concentrations), the percentage of water–derived oxygen into the product sulfate significantly increased and essentially all oxygen atoms were sourced from water (Table 4, Figure 3). The average δ^18^O_SO4_ vs. δ^18^O_H2O_ for all the experiments including both the lag phase and exponential growth phase reveal a slope of 0.87 ± 0.07 suggesting between 80 and 94% of sulfate-oxygen was derived from water. This indicates that we can consider water the sole source of oxygen atoms in the product sulfate during elemental sulfur oxidation by *A. thiooxidans* (Figure 3). This is consistent with previous studies that suggest that oxygen atoms in sulfate produced during biological oxidation of elemental sulfur are derived from water (Mizutani and Rafter, 1969; Balci et al., 2012; Table 4, Figure 3). The incorporation of atmospheric O_2_ into sulfate during the initial lag phase of the experiments might have resulted from the experimental design, which may have permitted chemisorption of O_2_ on sulfur surfaces and its further incorporation into initial sulfate, which was previously suggested during pyrite oxidation experiments (Tichomirowa and Junghans, 2009). As the experiment progresses and enters exponential growth phase, the incorporation of this chemisorbed O_2_ decreases and all oxygen atoms are derived from water.

**Table 4:** Oxygen and sulfur isotope composition and isotopic enrichment factors for sulfate produced from microbial oxidation of elemental sulfur.

**Time (hours)**	**W1 Experiments**			**W2 Experiments**			**W3 Experiments**			**% oxygen H_2_O^a^**	**ε^18^${\text{O}}_{\text{SO}4--\text{H}2\text{O}}^{\text{b}}$**
	**δ^18^O_SO4_ (‰)**	**δ^34^S_SO4_ (‰)**	**ε^34^S_SO4__–S0_ (‰)**	**δ^18^O_SO4_ (‰)**	**δ^34^S_SO4_ (‰)**	**ε^34^S_SO4__–S0_ (‰)**	**δ^18^O_SO4_ (‰)**	**δ^34^S_SO4_ (‰)**	**ε^34^S_SO4__–S0_ (‰)**		**(‰)**
4	n.d	17.01	−1.19	n.d	n.d	n.d	n.d	n.d	n.d	n.d	n.d
24	−4.2	16.4	−1.79	12.7/51.2^*^	17.12	−1.08	15.4/40.41^*^	n.d	n.d	58 ± 0.3	n.a
33	−6.73	n.d	n.d	12.5/34.7^*^	n.d	n.d	23.4/51.7^*^	n.d	n.d	66 ± 0.0	n.a
48	−10.1	16.7	−1.49	20.1/29.2^*^	17.41	−0.79	30/42.5^*^	16.5	−1.75	60 ± 1.0	n.a
60	−9.1	n.d	n.d	25.3/33.6^*^	n.d	n.d	45.6	n.d	n.d	63 ± 4.0	n.a
72	−10.8	17.2	−0.97	48.32	17.43	−0.77	52.1	n.d	n.d	76 ± 0.17	n.a
84	−11.0	n.d	n.d	54.3	n.d	n.d	72.4	17.3	−0.9	96 ± 6.0	−5.2 ± 3.9
96	−9.3	16.9	−1.23	56.2	17.20	−1.00	76.7	17.2	−1	98 ± 4.0	−3.7 ± 2.7
124	−11.2	n.d	n.d	54.4	n.d	n.d	78.3	17.4	−0.82	1.02 ± 0.0	−5.9 ± 0.9
146	−10.9	16.9	−1.28	57.6	17.40	−0.80	77.2	n.d	n.d	1.01 ± 0.0	−4.8 ± 3.7
168	−9.9	n.d	n.d	57.4	n.d	n.d	76.2	n.d	n.d	1.00 ± 0.0	−4.0 ± 3.3
264	−10.6	17.1	−1.09	52.7	17.35	−0.85	77.4	n.d	n.d	99 ± 0.0	−5.5 ± 0.07
336	−10.9	17.6	−0.56	56.2	17.69	−0.51	75.1	17.6	−0.6	99 ± 0.0	−5.0 ± 3.8
504	n.d	n.d	n.d	59.2	17.60	−0.62	n.d	n.d	n.d	n.d	n.d
720	−10.6	18.0	−0.13	57.5	17.73	−0.47	79.3	17.9	−0.35	1.03 ± 0.0	−4.7 ± 2.6
Average	−9.6/−10.2 (*n* = 13)	17.1 ± 0.2	−1.1 ± 0.2	50.2/51.4 (*n* = 14)	17.4 ± 0.2	−0.8 ± 0.2	64.9/65 (*n* = 13)	17.3 ± 0.2	−0.9 ± 0.2	87 ± 7.0^c^	−4.2 ± 4.4^c^
δ^18^O_H2O_initial_ (‰)	−4.44			58.4			84.4				
δ^18^O_H2O_final_ (‰)	−5.60			58.04			81.70				
Avg. δ^18^O_H2O_(‰)	−5.02			58.22			83.05				

δ^34^S_S0_ = 18.2 ± 0.2‰ (n = 5);

* Calculated values based on the mass balance equation (see text);

a Estimated from linear regressions between δ^18^O_SO4_ and δ^18^O_H2O_ (Balci et al., 2012);

b ε^18^O_SO4-H2O_ was obtained from the intercept calculated from linear regressions between δ^18^O_SO4_ and δ^18^O_H2O_ (Balci et al., 2012);

c Estimated from linear regressions between average δ^18^O_SO4_ (n = 37) and average δ^18^O_H2O_ (n = 3) values (Figure 3);

*n.a, not applicable; n.d, not determined*.

The δ^18^O of O_2_ in the flasks was +23.5 ± 0.5‰ (*n* = 4) at the beginning of study and +24.3 ± 1.7‰ (*n* = 4) at the end of the study suggesting that the δ^18^O of O_2_ was constant within analytical error over the duration of the experiments. Since both water and atmospheric O_2_ were incorporated into sulfate during the initial stage of elemental sulfur oxidation, determining ε^18^O_SO4−H2O_ and ε^18^O_SO4−O2_ and their relative contributions to the overall δ^18^O_SO4_ was not possible. Since all oxygen atoms in the product sulfate were derived from water during the main stage of the experiment, the ε^18^O_SO4−H2O_ can be calculated from the y-intercepts of δ^18^O_H2O_ vs. δ^18^O_SO4_ plots (Table 4, Figure 3). In this case, ε^18^O_SO4−H2O_ ranged from −5.9 to −3.7‰ with an average of −4.8‰ during the main stage oxidation experiments (84–720 h). An average ε^18^O_SO4−H2O_ of −4.2 ± 4.4‰ can be calculated when the δ^18^O_SO4_ values from all time points in the experiments are used (including those in the lag phase; Figure 3), meaning that ^16^O from water is preferentially incorporated into sulfate (normal isotope effect).

**Figure 4:**
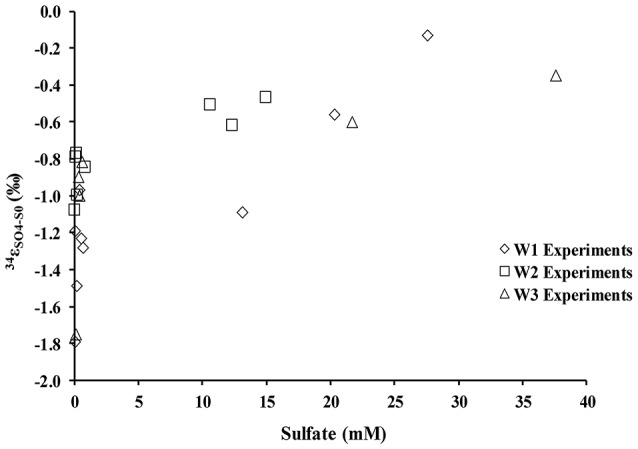
Sulfur isotope enrichment during oxidation of elemental sulfur in the presence of *A. thiooxidans*.

During the oxidation of elemental sulfur, the δ^34^S of the sulfate produced ranged from +16.4 to +18.0‰ with averages of 17.1, 17.4, and 17.3‰ for the W1, W2, and W3 experiments, respectively. Over the course of the experiments with elemental sulfur, the average difference between elemental sulfur and the sulfate formed (ε^34^S_SO4−S0_) was −1.1, −0.8, and −0.9‰ calculated from the W1, W2, and W3 experiments, respectively; the δ^34^S of the product sulfate is almost the same as the elemental sulfur substrate (Table 3). During the lag stage of the oxidation of elemental sulfur, a slightly larger normal sulfur isotope fractionation between elemental sulfur and sulfate was found (approximately −1.8‰; Table 4, Figure 4).

### Oxygen and sulfur isotopic composition of sulfate produced from oxidation of tetrathionate

**Figure 5:**
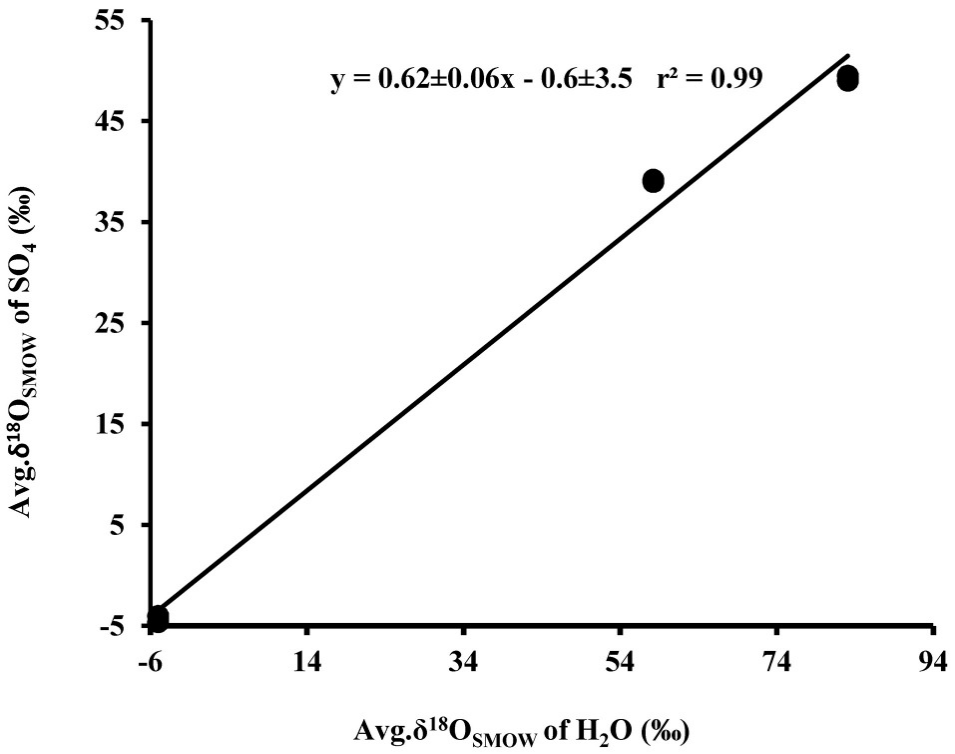
Plot of the average δ^18^O_SO4_ produced from biological (•) and abitoic (◦) oxidation of tetrathionate vs. average δ^18^O_H2O_ used in the experiments (see Table 4).

Plots of δ^18^O_SO4_ vs. δ^18^O_H2O_ for the experiments involving the oxidation of tetrathionate show a strong linear correlation (*r*^2^ > 0.99) with a slope of 0.62 ± 0.06 suggesting the incorporation of 62% water-derived oxygen into the sulfate with the remainder derived from atmospheric O_2_ or from tetrathionate (Figure 5). In contrast to the experiments with elemental sulfur, the percentage of water-derived oxygen into sulfate was lower and did not show significant change with the progressive oxidation from lag to exponential growth phase (Table 5).

**Table 5:** Oxygen and sulfur isotope composition and isotopic enrichment factors for sulfate produced from microbial oxidation of tetrathionate.

**Time (hours)**	**W1 Experiments H_2_O^a^**			**W2 Experiments**			**W3 Experiments**			**% oxygen**
	**δ^18^O_SO4_ (‰)**	**δ^34^S_SO4_ (‰)**	**ε^34^S_SO4−S4O6_ (‰)**	**δ^18^O_SO4_ (‰)**	**δ^34^S_SO4_ (‰)**	**ε^34^S_SO4−S4O6_ (‰)**	**δ^18^O_SO4_ (‰)**	**δ^34^S_SO4_ (‰)**	**ε^34^S_SO4−S4O6_ (‰)**	
48	−5.7	n.d	n.d	n.d	n.d	n.d	n.d	n.d	n.d	n.d
144	−4.2	7.3	3.4	40.1	9.2	5.3	22.3/51.9^*^	n.d	n.d	64
192	−4.4	9.4	5.5	39.2	8.1	4.2	33.1/47.2^*^	11.8	7.9	60
240	−2.6	8.9	4.9	42.3	7.81	3.9	49.2	n.d	n.d	61
288	−3.8	7.2	3.2	37.2	n.d	n.d	40.1	7.8	3.9	53
360	−4.1	6.2	2.2	42.5	6.8	2.9	n.d	n.d	n.d	n.d
408	−4.5	5.8	1.9	38.7	7.2	3.3	49.2	6.7	2.8	62
480	−4.4	6.1	2.2	34.6	6.6	2.7	47.4	n.d	n.d	59
600	−3.5	5.4	1.4	35.4	n.d	n.d	51.2	5.9	2.0	61
720	−2.6	6.4	2.4	38.8	5.9	2.0	52.2	6.1	2.2	62
Average	−4.0, −4.6 (*n* = 10)	6.9 ± 0.2	2.9 ± 0.2	38.8/39.2 (*n* = 9)	7.4 ± 0.2	3.5 ± 0.2	49/49.5 (*n* = 8)	7.7 ± 0.2	3.8 ± 0.2	62 ± 0.06^b^
δ^18^O_H2Oinitial_ (‰)	−4.44			58.4			84.4			
δ^18^O_H2Ofinal_ (‰)	−5.60			58.04			81.70			
Avg. δ^18^O_H2O_(‰)	−5.02			58.22			83.05			

δ^34^S_S4O6_ = 3.9‰ (n = 4);

a Estimated from linear regressions between δ^18^O_SO4_ and δ^18^O_H2O_ (Balci et al., 2012);

b Estimated from linear regressions between average δ^18^O_SO4_ (n = 27) and average δ^18^O_H2O_ (n = 3) values (Figure 4);

*n.d, not determined*.

**Figure 6:**
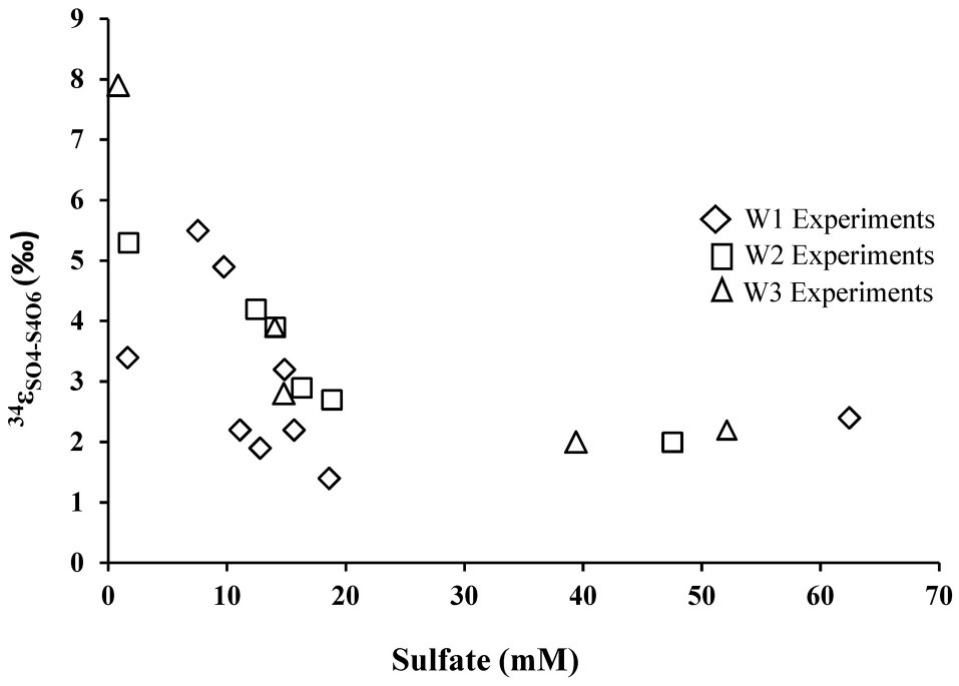
Sulfur isotope enrichment during oxidation of tetrathionate in the presence of *A. thiooxidans*.

During growth on tetrathionate, *A. thiooxidans* produced sulfate enriched in ^34^S compared to the δ^34^S of the tetrathionate substrate (which was 3.9‰). The calculated sulfur isotope fractionation (ε^34^S_SO4−S4O6_) was between +1.4 and +7.9‰ with an average of 2.9‰ (*n* = 9), 3.5‰ (*n* = 7), and 3.8‰ (*n* = 5) for the W1, W2, and W3 experiments, respectively (Table 5, Figure 6). The ε^34^S_SO4−S4O6_ was large during the initial stage of experiments, and became substantially smaller over time (Table 5, Figure 6), but remained inverse (^34^S preferentially ended up in sulfate).

## Discussion

### Microbial oxidation of tetrathionate and elemental sulfur

In all experiments with *A. thiooxidans*, the pH dropped from the initial pH to between 0.68 and 2.0 at the end of the month-long incubation, regardless if the substrate was elemental sulfur or tetrathionate (Figures 1, 2, Tables 2, 3). In the aseptic abiotic control experiments, the pH rose after 150 h from 4.1 to 4.2 with elemental sulfur, and remained invariant at 4.5 until 144 h following a decrease to 4.15 in the experiments with tetrathionate (Figures 1D, 2D). The amount of acid generation and sulfate produced are substrate dependent; the pH was lower and the amount of sulfate produced was higher in the experiments with elemental sulfur than for those with tetrathionate. The microbial disproportionation of tetrathionate caused a partial reduction of tetrathionate to thiosulfate and a fractional oxidation to sulfate and elemental sulfur via reaction (9) rather than a straight oxidation of tetrathionate to sulfate (reaction 7),

(9) $$\begin{array}{l} {\text{S}}_{4}{\text{O}}_{6}^{2-}+{\text{H}}_{2}\text{O}\to {\text{S}}_{2}{\text{O}}_{3}^{2-}+{\text{S}}^{0}+\text{S}{\text{O}}_{4}^{2-}+2{\text{H}}^{+} \end{array}$$

**Figure 7:**
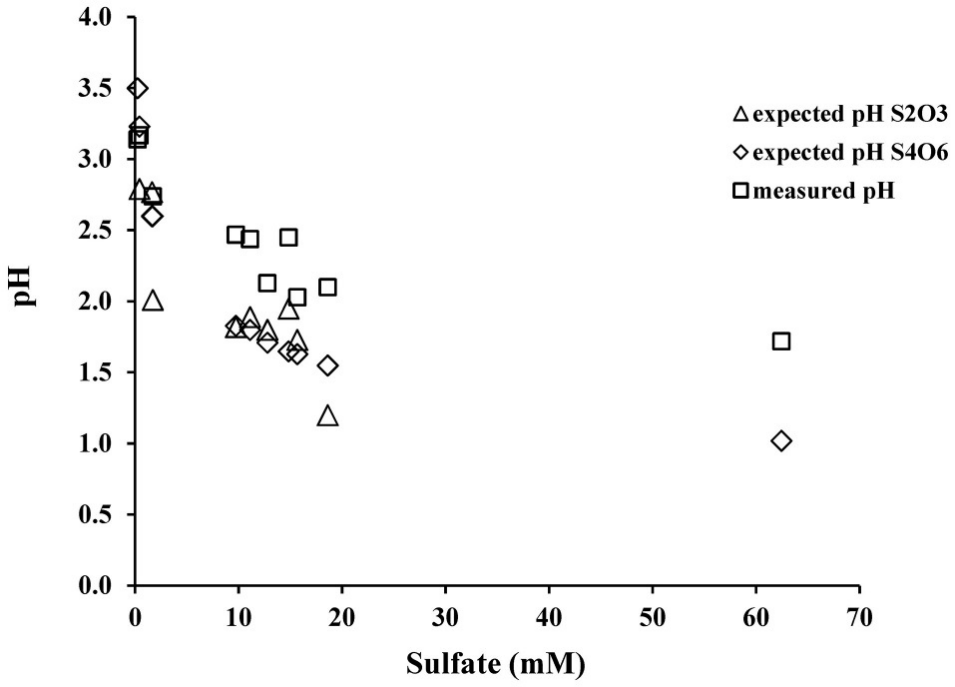
Relationship between sulfate and pH as predicted from the oxidation stoichiometry of tetrathionate (reaction 9) and thiosulfate (reaction 13) or as measured from the experiments.

This is supported by the fact that when *A. thiooxidans* grew on tetrathionate, there is less acidity generated and less sulfate produced, confirming that complete oxidation of tetrathionate to sulfate (reaction 7) does not occur (Figure 7). Our data also suggest that during tetrathionate oxidation the rates of the subsequent thiosulfate and elemental sulfur oxidation are lower than the rate of production of both species, leading to an accumulation of thiosulfate and elemental sulfur in the media (Figure 2).

The product thiosulfate, initially produced during both elemental sulfur and tetrathionate oxidation, may further be converted to sulfite and elemental sulfur through the initial cleavage of thiosulfate (reaction 10) followed by the oxidation of elemental sulfur (reaction 11) and sulfite to sulfate (reaction 12) (Meulenberg et al., 1993; Beard et al., 2011). The net reaction (13) is the overall oxidation of thiosulfate to sulfate.

(10) $$\begin{array}{l} {\text{S}}_{2}{\text{O}}_{3}^{2-}+2{\text{H}}^{+}\to {\text{S}}^{0}+{\text{H}}_{2}\text{S}{\text{O}}_{3} \end{array}$$

(11) $$\begin{array}{l} {\text{S}}^{0}+{\text{O}}_{2}+{\text{H}}_{2}\text{O}\to {\text{H}}_{2}\text{S}{\text{O}}_{3} \end{array}$$

(12) $$\begin{array}{l} 2{\text{H}}_{2}\text{S}{\text{O}}_{3}+{\text{O}}_{2}\to 2\text{S}{\text{O}}_{4}^{2-}+4{\text{H}}^{+} \end{array}$$

(13) $$\begin{array}{l} {\text{S}}_{2}{\text{O}}_{3}^{2-}+{\text{H}}_{2}\text{O}+2{\text{O}}_{2}\to 2\text{S}{\text{O}}_{4}^{2-}+2{\text{H}}^{+} \end{array}$$

When the pH of the experimental solution reached 2.0, thiosulfate was almost completely exhausted but small amounts of elemental sulfur remained and sulfate became the major anion (Figure 2, Table 3). If we combine the initial oxidation of tetrathionate to thiosulfate, and tetrathionate disproportionation to sulfate and elemental sulfur (reaction 6) with the overall oxidation of thiosulfate with O_2_ (reaction 11) we can predict the expected ratios of proton to sulfate generation during oxidation of tetrathionate as suggested by Bernier and Warren (2007). The predicted pH decrease from reactions (9) and (13) exceeds the observed pH decrease in our experiments indicating a substantial proton consumption occurred in parallel to the proton production during these oxidation reactions (Figure 6). Proton consumption during microbial oxidation of tetrathionate and thiosulfate has previously been reported under similar experimental conditions and attributed to microbial disproportionation reactions occurring during the oxidation of tetrathionate and thiosulfate (e.g., reaction 10, Bernier and Warren, 2007; Houghton et al., 2016). Reaction (14), which is microbially-mediated, has been suggested to consume protons under conditions similar to our laboratory experiments:

(14) $$\begin{array}{l} 2{\text{S}}_{2}{{\text{O}}_{3}}^{2-}+2{\text{H}}^{+}+0.5{\text{O}}_{2}\to {\text{S}}_{4}{{\text{O}}_{6}}^{2-}+{\text{H}}_{2}\text{O} \end{array}$$

Production of tetrathionate (up to 7.7 mM) from thiosulfate in experiments buffered at low pH has previously been reported (Houghton et al., 2016), which corroborates the hypothesis that reaction (14) plays an important role in buffering the pH. Bernier and Warren (2007) modeled the kinetics of the abiotic disproportionation of thiosulfate over a pH range of 1.5–4.0 according to the following reaction (reaction 15):

(15) $$\begin{array}{l} {\text{S}}_{2}{\text{O}}_{3}+2{\text{H}}^{+}\to {\text{S}}^{0}+{\text{H}}_{2}\text{S}{\text{O}}_{3} \end{array}$$

Their data indicated that this reaction was likely too slow to play a significant role; hence thiosulfate should not abiotically disproportionate to elemental sulfur and sulfite under experimental conditions similar to ours (Johnston and McAmish, 1973; Druschel et al., 2003). Lower concentrations of thiosulfate, sulfate and insignificant pH change measured in the abiotic experiments are consistent with these previous studies and further suggest that formation of thiosulfate and sulfate are due to microbial activity (Druschel et al., 2003; Bernier and Warren, 2007; Figure 2D).

Mass balance calculations demonstrate that up to 34% of the total sulfur is not accounted for by the sulfur species we measured (tetrathionate, thiosulfate, and elemental sulfur) during the experiment (Table 3). Sulfite is not expected to account for a significant fraction of the sulfur mass balance, since it should be rapidly oxidized under our experimental conditions (Pronk et al., 1990; Kappler and Dahl, 2001). This “missing sulfur” in the tetrathionate experiments may be linked to the formation of long-chain polythionates such as tri-, penta-, and hexa/thionate (Steudel et al., 1987; Pronk et al., 1990; Druschel et al., 2003; Bernier and Warren, 2007; Shiers et al., 2011). The production of long-chain polythionates has been also reported in aerated *A. ferrooxidans* suspensions, which is phylogenetically similar (at the genus level) to *A. thiooxidans*, incubated with tetrathionate (Steudel et al., 1987; Hazeu et al., 1988). Druschel et al. (2003) attributed the formation of trithi-and pentathionate to reactions involving polysulfane monosulfonic acids (Steudel et al., 1987, 1988; Pronk et al., 1990; Steudel, 2003). As suggested by Druschel et al. (2003) the chemical reactions involving polysulfane monosulfonic acids may have been altered the oxidation stoichiometry of tetrathionate and thiosulfate (reactions 9 and 13, respectively) resulting in a change in the expected stoichiometric ratio between sulfate and acid. Another possibility is that volatile sulfur species escape the experiments. Such a process has been considered for the initial stage of pyrite oxidation in acidic solutions, where it was showed that under highly acidic conditions, sulfite can form sulfur dioxide gas (SO_2_), and degas into the headspace (Brunner et al., 2008). The conversion of sulfite into SO_2_ consumes protons, i.e., which would slow down the drop to lower pH during tetrathionate oxidation.

When elemental sulfur is the sole substrate or is produced as a by-product of tetrathionate disproportionation (reactions 9 and 10), it needs to be activated before it is transferred into the periplasm of microorganisms for further chemical reaction (Bobadilla Fazzini et al., 2013). Following its activation, the first step of elemental sulfur oxidation is thought to be the transition of sulfur to thiosulfate catalyzed by sulfur dioxygenase (SDO), which generates sulfite (Meulenberg et al., 1993; Bobadilla Fazzini et al., 2013). The activation of elemental sulfur results in a slower growth and lower cell numbers represented by a long lag phase during the oxidation reactions, as discussed above (Figure 1). Trace amount of sulfite detected in the experiments suggest that sulfite may have been produced but was readily converted to sulfate (via reactions 9, 10, and 11). The formation and subsequent oxidation of sulfite to sulfate occurs during tetrathionate and elemental sulfur oxidation in the presence of various *Acidithiobacillus* spp., as has previously been suggested (Hallberg et al., 1996; Suzuki, 1999; Shiers et al., 2011).

### Fractionation of sulfur isotopes between sulfate, elemental sulfur, and tetrathionate

There was a negligible sulfur isotope fractionation observed during the microbial oxidation of elemental sulfur (Table 4, Figure 4). However, a small but significant sulfur isotope fractionation (ε^34^S_SO4−S0_ ~−2‰) was observed during the initial 48 h of the experiment. This change in sulfur isotope fractionation over the course of the experiment has been previously reported for experiments with a phylogenetically similar microorganism, *A. ferrooxidans* (Balci et al., 2012) and in earlier studies exploring elemental sulfur oxidation (Kaplan and Rittenberg, 1964; Mizutani and Rafter, 1969; McCready and Krouse, 1982). This reported larger sulfur isotope fractionation in the initial stage of microbial growth might relate to sulfur isotope fractionation during the activation of elemental sulfur. This activation stage requires an opening of the S_8_ ring and thus a bond-breaking processes between S–S atoms by the thiol groups of cysteine residues (Tichomirowa and Junghans, 2009). Overall, our results demonstrate that elemental sulfur is fully oxidized to sulfate under acidic conditions, resulting in an overall negligible sulfur isotope fractionation, a finding that is consistent with the fact that sulfur isotope fractionation associated with oxidation of solid phase sulfur is insignificant relative to the oxidation of aqueous hydrogen sulfide (Nakai and Jensen, 1964; McCready and Krouse, 1982; Taylor et al., 1984; Fry et al., 1986; Balci et al., 2007, 2012; Thurston et al., 2010; Smith et al., 2012).

In contrast to the elemental sulfur experiments, biological oxidation of tetrathionate produced sulfate that is enriched in ^34^S relative to tetrathionate throughout the experiments. The average ε^34^S_SO4−S4O6_ of +2.9, +3.5, and +3.8‰ obtained from the experiments in this study falls with the range of other experimental results for the microbial oxidation of various sulfur compounds (Table 1). However, it must be noted the magnitude of this inverse sulfur isotope fractionation decreased over the course of the experiment, from approximately to +7.9 to +1.4‰ (Table 5, Figure 6). The oxidation of tetrathionate to sulfate involves a series of microbially-catalyzed reactions (given above, reactions 9–14). The sulfur isotope fractionation associated with multi-step microbial transformations have not been fully determined, and there is a large gap in knowledge for reactions taking place at low pH. Previous studies on microbially-catalyzed sulfur oxidation have produced vastly different results that may be difficult to reconcile. For example, a large inverse sulfur isotope fractionation (ε^34^S_SxO6−S2_ = +0.6 to +19‰) was observed during the oxidation of sulfide to polythionates by *A. thiooxidans*, whereas, a large normal sulfur isotope fractionation (−18 to −10.5‰) was observed with *A. thiooxidans* oxidizing sulfide to sulfate (Kaplan and Rittenberg, 1964). It is likely that two mixed-valence state sulfur species, thiosulfate and tetrathionate, play a critical role in shaping the isotope composition of the product of the oxidation process. Thiosulfate and tetrathionate possess sulfur atoms with different valence states, whereby the sulfane (S−) sulfur atoms (connected by S–S bonds) have a lower valence state than the sulfonate (−SO_3_) sulfur species, which have a S–S bonds and three S–O bonds (Druschel et al., 2003). The sulfur isotope difference between the two sulfur atoms in thiosulfate is estimated to be between 6 and 14‰ with the sulfonate sulfur species enriched in ^34^S relative to the sulfane species (Chambers and Trudinger, 1979; Fry et al., 1986; Smock et al., 1998). In analogy, it is likely that at chemical equilibrium, the sulfonate species of tetrathionate are substantially enriched in ^34^S relative to the sulfane species. It can thus be argued that the shift from large inverse sulfur isotope fractionation (+7.9‰) to a smaller sulfur isotope fractionation of +1.4‰ is due to the initial disproportionation of tetrathionate into sulfate (reaction 9), followed by a delayed oxidation of other sulfur intermediates to sulfate. If the former sulfate is mainly supplied from isotopically heavy sulfonate-sulfur, and the latter from isotopically light sulfane-sulfur, one would expect to observe the described trend. We did not analyze the sulfur isotope composition of the sulfane and sulfonate moieties of the tetrathionate used in our experiments, and therefore, cannot further test this hypothesis, however, the oxygen isotope signature of formed sulfate provides additional insight.

### Fractionation of oxygen isotopes between SO_4_ and H_2_O during oxidation of elemental sulfur and tetrathionate

The contribution of water-derived oxygen to sulfate during oxidation of elemental sulfur by *A. thiooxidans* ranged from 58% to 103% and the ε^18^O_SO4−H2O_ was estimated to be between −5.9 ± 0.9 to −3.7 ± 2.7‰ with a mean of −4.8 ± 3.2‰ (Table 4). The high percentage of sulfate-oxygen derived from water is consistent with previous studies on microbially-mediated elemental sulfur oxidation (Balci et al., 2012; Smith et al., 2012) indicating similar elemental sulfur processing pathways among *Acidithiobacillus* spp.

There are two reasons why oxygen from O_2_ is negligible in sulfate formed from elemental sulfur oxidation. First, biological oxidation of solid elemental sulfur proceeds via stepwise enzymatic reactions where molecular oxygen acts as an electron acceptor (Kelly, 1982; Pronk et al., 1990). The first documented step in sulfur oxidation is the activation of extracellular elemental sulfur (S_8_) to thiol-bound sulfane sulfur atoms (R-S-SH) and then it is transferred into the periplasm where it is oxidized by the sulfur dioxygenase (SDO) to produce sulfite which further combines chemically with sulfur atoms to produce thiosulfate (Pronk et al., 1990; Suzuki, 1999; Bobadilla Fazzini et al., 2013; Yin et al., 2014). The electron transport system for elemental sulfur oxidation is the likely cause for the complete incorporation of water oxygen into sulfate. *Acidithiobacillus* spp., derive energy from sulfur oxidation by coupling to reduction of oxygen from O_2_, but the electrons from elemental sulfur pass through several steps of the electron transport system before they reduce O_2_ to water in the final step. Consequently, the reduction of O_2_ to water is physically separated from the final oxidation of sulfite to sulfate and the oxygen in sulfate then can derive wholly from water (Kelly, 1982). The transfer of electrons from sulfur to the electron acceptor (O_2_) provides energy by cycling electrons through the electron transport system (Ehrlich, 1982). From this follows that if the bacteria produce sulfate through the electron transport system then the δ^18^O_SO4_ of the produced sulfate should not be affected by δ^18^O_O2_.

Secondly, oxygen isotope exchange between sulfite, a common intermediate valence state sulfur species, and water is likely to occur under the low pH of our experimental conditions and may be partially responsible for the large calculated water-oxygen incorporation into sulfate (reactions 10–12). It has been reported that oxygen isotope exchange between sulfite and water is on the order of nanoseconds under acidic conditions, which is in stark contrast to the multi-million year timescale for oxygen isotope exchange between sulfate and water at circum-neutral pH (Lloyd, 1968; Pearson and Rightmire, 1980; Holt et al., 1981). Chiba and Sakai (1985) reported that oxygen isotope exchange between sulfate and water should take ~10^9^ year at 100–300°C and pH 2 to 7—based on laboratory experiments. Extrapolating the experimental data of Hoering and Kennedy (1957) and Chiba and Sakai (1985) to pH 0, 1, and 2, the respective half times required for oxygen isotopic exchange between sulfate and water could be as low as 1 year at pH 0 and 10^5^ years at pH 2 (Rennie and Turchyn, 2014). Based on these results, it can be safely assumed that sulfate–water oxygen isotope exchange is unlikely to occur under our experimental conditions. Alternatively, Mizutani and Rafter (1969, 1973), and Fritz et al. (1973) demonstrated that oxygen isotope exchange between sulfate and water proceeds through enzyme-bound intermediates during the bacterial reduction of sulfate. Similar enzyme-mediated oxygen isotope exchange processes might occur during mixed-valence state sulfur species oxidation to sulfate.

The enrichment in ^18^O in sulfite during oxygen isotope equilibration with water (ε^18^O_SO3−H2O_) has been addressed in several studies, but large uncertainties remain. Holt et al. (1981) reported that δ^18^O_SO3_ was enriched by 24‰ with respect to δ^18^O_H2O_ under equilibrium conditions. Consistent with Holt et al. (1981) and Brunner et al. (2006) reported that ε^18^O_SO3−H2O_ increased as pH decreased and reported ε^18^O_SO3−H2O_ of 11.5 and 7.9‰ for pH 7.2 and 8, respectively at 23°C. Since no experiments have been conducted for strongly acidic pH it is unsure if ε^18^O_SO3−H2O_ would continue to increase with decreasing pH (Müller et al., 2013a; Wankel et al., 2014). Several estimates for ε^18^O_SO4−H2O_ have been reported during oxidation of reduced sulfur compounds. For example, Smith et al. (2012) estimated the ε^18^O_SO4−H2O_ ranging −6.2 to −0.9‰ during elemental sulfur oxidation by *A. thiooxidans* under various temperature and nutrient regimes while Mizutani and Rafter (1969) found an oxygen isotope fractionation of 0‰. The range of −5.9 ± 0.9 to −3.7 ± 2.7‰ for ε^18^O_SO4−H2O_ from the current experiments appears to be consistent with the range reported by these studies.

In contrast to experiments with elemental sulfur, our data demonstrate that contribution of water-derived oxygen to sulfate ranged from 53 to 64% (average of 62%) during oxidation of tetrathionate (Tables 3, 5, Figure 5), which is at the lower end of the percentage of water-derived oxygen ranging between 50 and 97% reported for biological and abiological sulfide oxidation to sulfate under aerobic and acidic conditions (Toran and Harris, 1989; van Stempvoort and Krouse, 1994; Balci et al., 2007, 2012; Table 1). It is interesting to note that 37.5% of oxygen in sulfate could be derived from tetrathionate (reaction 7), which leaves 62.5% to oxygen from water or O_2_. In analogy to the findings for elemental sulfur oxidation, it could be inferred that no oxygen from O_2_ is incorporated into sulfate during tetrathionate oxidation. Such an interpretation would demand that the entire oxygen inventory of tetrathionate ends up in sulfate, which requires that no oxygen-atoms from tetrathionate are exchanged with oxygen from water, i.e., no S–O bonds from the sulfonate moieties are broken. While this interpretation is appealing, it is difficult to reconcile it with the argument that the shift from large inverse sulfur isotope fractionation to a smaller sulfur isotope fractionation is due to the initial transformation of the sulfonate moiety of tetrathionate into sulfate followed by a delayed oxidation of other sulfur intermediates that originated from the sulfane moiety of tetrathionate. If this was the case, one would expect to initially observe a minimal contribution of water-oxygen (i.e., 25%, as fourth oxygen atom required to convert sulfonate into sulfate), followed by an increase toward 62.5% concomitant to the oxidation of increase in the oxidation of sulfur intermediates derived from the sulfane moiety of tetrathionate. This is not the case, as there is no trend that would indicate a consistent increase in the contribution of oxygen from water to sulfate over time (Table 5). This indicates that at least one of the two proposed explanations—either incorporation of oxygen from water and none from air, or a change in the observed sulfur isotope fractionation—is overly simplistic. Since oxygen isotopes are likely fractionated during the incorporation of oxygen from water, tetrathionate, and molecular O_2_ into the product sulfate, it was not possible to directly determine the corresponding isotope fractionation factors ε^18^O_SO4−H2O_, ε^18^O_SO4−S4O6_, and ε^18^O_SO4−O2_ (Sessions and Hayes, 2005).

### Implications for the natural environment

Recent experimental studies have shown the formation of mixed-valence-state sulfur species during metal sulfide oxidation in acid-mine drainage settings and suggested that these sulfur species play a significant role in redox sulfur cycling in these environments (Schippers et al., 1996; Druschel et al., 2003; Heidel and Tichomirowa, 2010). Therefore, while the occurrence of mixed-valence sulfur compounds has been reported in various environments (e.g., mine wastes, marine, and freshwater sediments), the role of these sulfur molecules and their contribution to the acidic redox cycling of sulfur remains unclear, particularly whether there may be a way to track the cycling of these species using stable isotopes. Most of our knowledge about how sulfur is reduced and re-oxidized derives from the sulfur and oxygen isotope composition of sulfate generated during sulfide mineral oxidation (biotic or abiotic; Taylor et al., 1984; Balci et al., 2007, 2012; Brunner et al., 2008; Balci, 2010; Heidel and Tichomirowa, 2010; Thurston et al., 2010; Sanliyuksel Yucel et al., 2016).

In general, a small sulfur isotope fractionation between metal sulfide and the product sulfate resulting from oxidation of sulfur under acid conditions by microorganisms has been reported (Table 1—Taylor et al., 1984; Rye et al., 1992; Seal, 2003; Balci et al., 2007; Heidel et al., 2009; Heidel and Tichomirowa, 2010; Thurston et al., 2010). Furthermore, only a small sulfur isotope fractionation generally accompanies the aerobic oxidation of H_2_S, S^0^, S_2_${\text{O}}_{3}^{2-}$, and ${\text{SO}}_{3}^{2-}$ to either S^0^ or ${\text{SO}}_{4}^{2-}$ (Fry et al., 1986; Table 1). In contrast, larger sulfur isotope fractionation has been found during disproportionation of S_2_${\text{O}}_{3}^{2-}$, S^0^, and ${\text{SO}}_{3}^{2-}$ at more neutral conditions (Habicht et al., 1998; Poser et al., 2016). Consistent with previous studies, the sulfur isotope fractionation of elemental sulfur oxidation to sulfate under aerobic acidic conditions was characterized by a small sulfur isotope fractionation in this study (Table 1). In contrast, we found significantly ^34^S-enriched sulfate during experiments involving tetrathionate oxidation under acid conditions, and observed that the incorporation of oxygen from water in this step is only 62%. Tetrathionate is accepted as a key intermediate in the oxidation of acid-insoluble sulfides, such as pyrite (e.g., Schippers and Sand, 1999; Druschel et al., 2003), a process that commonly displays little to no sulfur isotope fractionation, and often oxygen incorporation from water near to 100%, but with considerable scatter for both parameters (Table 1).

Our results suggest that if tetrathionate is produced in acidic conditions such as those found in acid-mine drainage, there is higher potential to observe such a scatter than under conditions where elemental sulfur or metal sulfide are directly oxidized to sulfate, with insignificant sulfur isotope fractionation (Table 1). The question becomes if there are conditions that would favor the expression of sulfur and oxygen isotope effects tied to the presence of tetrathionate. In the case of sulfur isotope fractionation, a prerequisite is that the sulfur from tetrathionate is not quantitatively converted to sulfate. Our microbial experiments show that tetrathionate oxidation is indeed not quantitative. According to Schippers (2004), the degradation of tetrathionate strongly depends on pH and the presence of catalysts such as pyrite. In acidic conditions, tetrathionate formed in the presence of pyrite is quickly hydrolyzed to disulfane-monosulfonic acid rather than oxidized to sulfate (Steudel et al., 1987; Schippers et al., 1996). Disulfate-monosulfonic acid is unstable and will react to form to various sulfur compounds (e.g., trithionate, pentathionate) before forming sulfate. The competition between microbial agents and mineral surfaces in the catalysis of tetrathionate oxidation may be decisive for fate of the sulfur moieties of tetrathionate, and control if the oxidation process is quantitative (no sulfur isotope fractionation) or incomplete (potential for sulfur isotope fractionation).

Microbes may also be key in catalyzing sulfur isotope exchange between sulfur moieties in tetrathionate or thiosulfate, or through reversibility of enzymatic steps, between different sulfur intermediates. For example, sulfur isotope exchange between the two sulfur atoms of thiosulfate leading to an enrichment of ^34^S in sulfate up to 12‰ during disproportionation of thiosulfate has been previously suggested (Habicht et al., 1998). The initial steps during tetrathionate oxidation, such as the transport into the cell without a change in its oxidation state, do not involve the breaking of bonds and should not generate sulfur isotope fractionation. However, in the periplasm the tetrathionate can be disproportionated to thiosulfate, elemental sulfur and sulfate by a tetrathionate hydrolase enzyme, such as the ones that have been isolated from acidophilic cells of *A. ferrooxidans* and *A. thiooxidans* (Meulenberg et al., 1992; De Jong et al., 1997; Yin et al., 2014). Tetrathionate disproportionation consists of multiple enzymatic steps that involve breaking of bonds and thus the sulfur isotopic fractionation measured between the final product sulfate and the reactant tetrathionate will reflect the sum of sulfur isotope fractionations associated with these enzymatic reactions. It is well documented that sulfur isotope fractionation during disproportionation may be controlled by the cell-specific rate of disproportionation in addition to various environmental and physicochemical factors (Habicht et al., 1998; Poser et al., 2016). During disproportionation of sulfur substrates such as elemental sulfur, thiosulfate, or tetrathionate, reversible reactions are involved that further modulate the sulfur isotope fractionation (Habicht et al., 1998; Kelly, 2008). Oxidation of thiosulfate from the reductive branch of tetrathionate disproportionation may also contribute to the larger overall sulfur isotope fractionation measured in our experiments. The co-occurrence of a larger sulfur isotope fractionation and the production of thiosulfate in this study suggests that there is a link between thiosulfate cycling and sulfur isotope partitioning (Table 3).

These considerations highlight that the formation of tetrathionate could be the reason why there is considerable scatter in the isotope data among various studies of the oxidation of pyrite and other reduced sulfur species. If the role of tetrathionate in these processes is understood, the scatter in the data sets could be used to decipher specific environmental conditions of different AMD systems based on sulfur and oxygen isotope signatures that are preserved in sulfate. Our pilot study demonstrates that tetrathionate clearly holds the potential to be a key compound in shaping the δ^34^S_SO4_ and δ^18^O_SO4_ of sulfate in AMD, and sets the stage for further investigations of the oxidation of this compound, such as the determination of the contribution of O_2_ to the formed sulfate, and experiments that test if the presence of ferric or ferrous iron, in combination with microorganism that catalyze iron oxidation, has an impact on the quantity of “missing sulfur” during tetrathionate oxidation.

## Conclusions

Sulfur isotope fractionation during the microbial oxidation of elemental sulfur to sulfate was negligible, while for the microbial oxidation of tetrathionate to sulfate the sulfur isotope fractionation was +3.5‰. Such a large sulfur isotope fractionation requires that ^34^S-depleted sulfur compounds form, which we attribute to the up to 34% of “missing sulfur” that was noticed in the experiments involving tetrathionate oxidation, which we hypothesize comprised tri/pentathionates, but also loss of sulfur dioxide to the atmosphere may contribute to the observed isotope fractionation. The δ^18^O of sulfate produced from the oxidation of elemental sulfur suggests that water-oxygen was the sole source of oxygen atoms for the sulfate ion, while the sulfate produced from tetrathionate oxidation derived oxygen from both water (62%). There are substantial differences in the sulfur and oxygen isotope signatures, acid generation and associated sulfur speciation between the oxidation of tetrathionate and elemental sulfur with the very same organism, an observation that likely can be generalized for other substrates in sulfur oxidation. Mixed-valence state sulfur species are microbially available and play substantial roles in sulfur cycle in acid-mine drainage systems. The analysis of both sulfur and oxygen isotopes in sulfate can be an important tool to detect and monitor the oxidation of such mixed-valence state sulfur species by *A. thiooxidans* in acidic environments.

## Author contributions

All authors listed have made a substantial, direct and intellectual contribution to the work, and approved it for publication.

### Conflict of interest statement

The authors declare that the research was conducted in the absence of any commercial or financial relationships that could be construed as a potential conflict of interest.
